# HOIL‐1 ubiquitin ligase activity targets unbranched glucosaccharides and is required to prevent polyglucosan accumulation

**DOI:** 10.15252/embj.2021109700

**Published:** 2022-03-11

**Authors:** Ian R Kelsall, Elisha H McCrory, Yingqi Xu, Cheryl L Scudamore, Sambit K Nanda, Paula Mancebo‐Gamella, Nicola T Wood, Axel Knebel, Stephen J Matthews, Philip Cohen

**Affiliations:** ^1^ MRC Protein Phosphorylation and Ubiquitylation Unit School of Life Sciences University of Dundee Dundee UK; ^2^ Cross‐Faculty NMR Centre Department of Life Sciences Imperial College London London UK; ^3^ Exepathology Exeter UK

**Keywords:** glycogen, polyglucosan, RBCK1, RBR E3 ligase, ubiquitination, Metabolism, Molecular Biology of Disease, Post-translational Modifications & Proteolysis

## Abstract

HOIL‐1, a component of the linear ubiquitin chain assembly complex (LUBAC), ubiquitylates serine and threonine residues in proteins by esterification. Here, we report that mice expressing an E3 ligase‐inactive HOIL‐1[C458S] mutant accumulate polyglucosan in brain, heart and other organs, indicating that HOIL‐1’s E3 ligase activity is essential to prevent these toxic polysaccharide deposits from accumulating. We found that HOIL‐1 monoubiquitylates glycogen and α1:4‐linked maltoheptaose *in vitro* and identify the C6 hydroxyl moiety of glucose as the site of ester‐linked ubiquitylation. The monoubiquitylation of maltoheptaose was accelerated > 100‐fold by the interaction of Met1‐linked or Lys63‐linked ubiquitin oligomers with the RBR domain of HOIL‐1. HOIL‐1 also transferred pre‐formed ubiquitin oligomers to maltoheptaose *en bloc*, producing polyubiquitylated maltoheptaose in one catalytic step. The Sharpin and HOIP components of LUBAC, but not HOIL‐1, bound to unbranched and infrequently branched glucose polymers *in vitro,* but not to highly branched mammalian glycogen, suggesting a potential function in targeting HOIL‐1 to unbranched glucosaccharides in cells. We suggest that monoubiquitylation of unbranched glucosaccharides may initiate their removal from cells, preventing precipitation as polyglucosan.

## Graphical abstract

Linear ubiquitin chain assembly complex (LUBAC) targeting and ubiquitin attachment to unbranched glucose polymers may prevent formation of toxic polyglucosan deposits in cells.

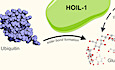

## Introduction

Mutations that reduce the expression of HOIL‐1 (haem‐oxidised IRP2 ubiquitin ligase‐1), also called RBCK1 (RING‐B‐Box‐coiled‐coil protein interacting with PKC 1 or RANBP2‐type and C3HC4‐type zinc finger‐containing protein 1), cause immuno‐insufficiency in mice (Tokunaga *et al*, 2009; MacDuff *et al*, 2015) and both auto‐inflammation and immuno‐insufficiency in humans (Boisson *et al*, 2012; Phadke *et al*, 2020). However, HOIL‐1 deficiency in humans also leads to cardiomyopathy and death from heart failure in early adulthood (Boisson *et al*, 2012; Nilsson *et al*, 2013; Wang *et al*, 2013; Fanin *et al*, 2015; Krenn *et al*, 2018; Phadke *et al*, 2020), which is unrelated to the immune defects, and arises from the progressive accumulation of toxic polyglucosan bodies in cardiac muscle and other tissues, such as the brain, with some patients also displaying cognitive impairment (Phadke *et al*, 2020; Chen *et al*, 2021). Mice expressing low levels of HOIL‐1 (Fujita *et al*, 2018) also form toxic polyglucosan bodies in cardiac muscle (MacDuff *et al*, 2015), brain and spinal cord (Nitschke *et al*, 2022), but it is the brain that is affected predominantly in mice, the animals displaying defects in learning, memory and motor coordination (Nitschke *et al*, 2022).

Polyglucosan bodies are dense inclusions of starch‐like polysaccharide that are insoluble because they lack the α1:6 branch points found in glycogen. Consequently, they have defective metabolism compared to glycogen and are resistant to digestion with α‐amylases (Hedberg‐Oldfors & Oldfors, 2015). The frequency of the α1:6 branch points, which occur after about every 12 α1:4‐linked glucose units, determines the topology, structure and solubility of glycogen. Mutations in glycogen‐metabolising enzymes cause a variety of glycogen storage diseases characterised by aberrant glycogen deposits (Kanungo *et al*, 2018).

HOIL‐1 is a component of the linear ubiquitin chain assembly complex (LUBAC) (Kirisako *et al*, 2006; Liu & Pan, 2018; Dittmar & Winklhofer, 2020). It is well established that this trimeric complex, comprising HOIL‐1, HOIP (HOIL‐1‐interacting protein) and Sharpin (Shank‐associated RH domain interactor), is required in signal transduction pathways that generate inflammatory mediators or lead to cell death (Gerlach *et al*, 2011; Ikeda *et al*, 2011; Tokunaga *et al*, 2011; Ikeda, 2015; Sasaki & Iwai, 2015; Shimizu *et al*, 2015). In these pathways, Met1‐linked ubiquitin (M1‐Ub) oligomers generated by the E3 ligase HOIP interact with several proteins that regulate innate immunity, such as the NEMO regulatory subunit of the canonical IκB kinase (IKK) complex. This leads to IKK activation and the phosphorylation and activation of its substrates, which include the transcription factors NF‐κB and IRF5 (Scheidereit, 2006; Lopez‐Pelaez *et al*, 2014; Ren *et al*, 2014) that stimulate expression of the mRNAs encoding many inflammatory mediators.

Like HOIP, HOIL‐1 is a member of the “RING in‐between RING” (RBR) subfamily of E3 ubiquitin ligases but, unlike HOIP, it does not generate Met1‐linked ubiquitin oligomers. Instead, it catalyses the attachment of ubiquitin to serine and threonine residues in proteins, forming ester bonds, and its substrates include components of Myddosomes that have critical roles in inflammatory mediator production (Kelsall *et al*, 2019b). The failure to generate ester‐linked ubiquitin chains in knock‐in mice expressing the E3 ligase‐inactive HOIL‐1[C458S] mutant can enhance or reduce the production of inflammatory cytokines, depending on the ligand, receptor and immune cell type (Petrova *et al*, 2021).

Since HOIL‐1 ubiquitylates the hydroxyl side chains of serine and threonine residues (Kelsall *et al*, 2019b; Rodriguez Carvajal *et al*, 2021), we hypothesised that it might also ubiquitylate hydroxyl groups in glucose and so be able to ubiquitylate glycogen directly. Here, we report that HOIL‐1 does indeed ubiquitylate glycogen and the smaller model oligosaccharide maltoheptaose *in vitro* and identify the site of ubiquitylation. Based on these and other unexpected findings reported in this study, we suggest a new role for HOIL‐1 and LUBAC in the ubiquitylation of unbranched glycogen molecules that may initiate their elimination from cells before they precipitate as toxic polyglucosan deposits.

## Results

### Polyglucosan accumulates in the brain and heart of HOIL‐1[C458S] mice

**Figure 1:**
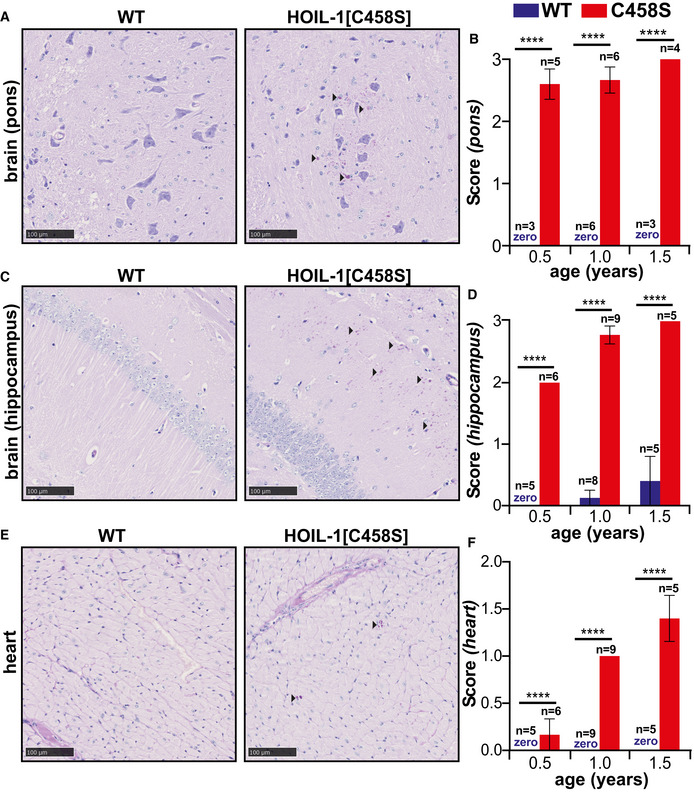
Deposition of α‐amylase‐resistant polyglucosan deposits in the hind brain and heart of HOIL‐1[C458S] mice A–FRepresentative PAS‐stained sections of the pons (A) and hippocampal (C) regions of the brain and heart (E) of 1‐year‐old HOIL‐1[C458S] and WT mice are shown. Scale bar = 100 μm. Arrow heads indicate α‐amylase‐resistant PAS‐positive polyglucosan deposits. Graphs showing α‐amylase‐resistant PAS scores of the pons (B) and hippocampal (D) regions of the brain and the heart (F) of HOIL‐1[C458S] (red) and WT (blue) mice aged 0.5, 1.0 and 1.5 years. The number of biological replicates analysed at each age is indicated. The word zero highlighted in blue indicates that no α‐amylase‐resistant, PAS‐positive material could be detected in the WT mice. The error bars show mean ± SEM. Statistical significance between the genotypes was calculated by using two‐way ANOVA and Šidák’s multiple comparison’s test. **** denotes *P* < 0.0001.

HOIL‐1 knockout (KO) mice exhibit early embryonic lethality (Fujita *et al*, 2018; Peltzer *et al*, 2018), but HOIL‐1 “KO” mice expressing low levels of a truncated protein comprising the N‐terminal ubiquitin‐like (UBL) and Npl4 zinc finger (NZF) domains, but lacking the C‐terminal region including the catalytic RBR domain (Fujita *et al*, 2018), accumulate polyglucosan in their brain, spinal cord and heart (MacDuff *et al*, 2015; Nitschke *et al*, 2022). Because HOIL‐1 stabilises its binding partner HOIP, the expression of HOIP and Sharpin is also greatly reduced in these HOIL‐1 “KO” mice. It was therefore unclear whether the accumulation of polyglucosan was caused by loss of the E3 ligase activity of HOIL‐1, loss of the E3 ligase activity of HOIP or reduced expression of the non‐catalytic domains of HOIL‐1, HOIP or Sharpin. In contrast, HOIL‐1, HOIP and Sharpin are expressed at normal levels in the E3 ligase‐inactive HOIL‐1[C458S] knock‐in mice that we have described previously (Kelsall *et al*, 2019b). We therefore investigated whether polyglucosan was present in the tissues of these mice. We found that polyglucosan did indeed accumulate in the brain of the HOIL‐1[C458S] mice, but not in their wild‐type littermates. Particularly high levels were present in the hind brain (pons) and the hippocampus, the extent of deposition being similar in mice varying in age from 0.5 to 1.5 years (Fig 1A–D). Similar to those found in human patients harbouring mutations in HOIL‐1 (Laforet *et al*, 2017), the polyglucosan deposits in the hippocampus and pons of HOIL‐1[C458S] mice were smaller and more speckled in appearance than those observed in other glycogenoses, such as those resulting from catastrophic general errors in glycogen synthesis, like the absence of glycogen branching enzyme.

**Figure EV1:**
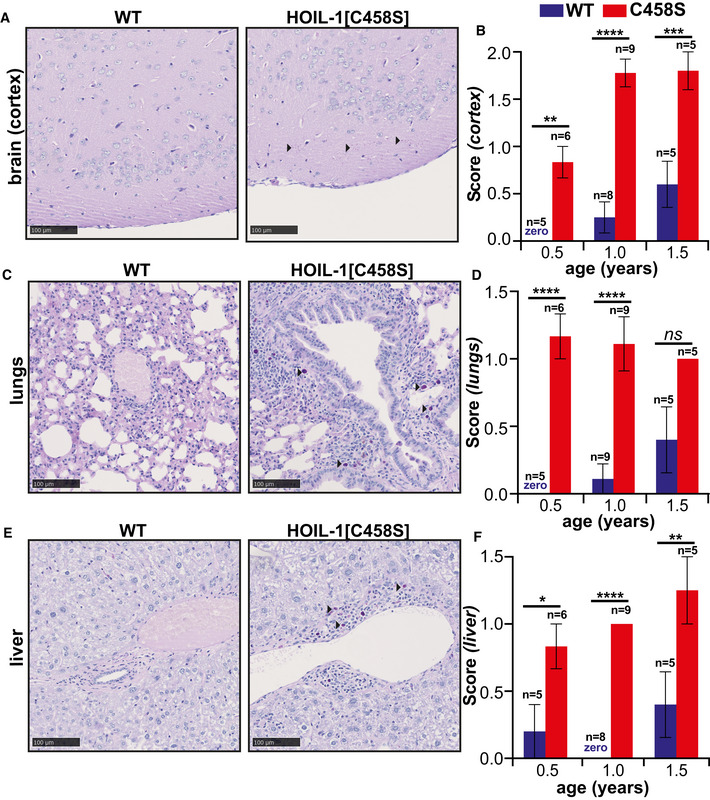
Deposition of α‐amylase‐resistant polyglucosan deposits in the brain cortex, lungs and liver of HOIL‐1[C458S] mice A–FRepresentative PAS‐stained sections of the brain cortex (A), lung (C) and liver (E) of 1‐year‐old HOIL‐1[C458S] and WT mice are shown. Scale bar = 100 μm. Arrow heads indicate α‐amylase‐resistant PAS‐positive polyglucosan deposits. Graphs quantitating α‐amylase‐resistant PAS scores of the brain cortex (B), lung (D) and liver (F) of HOIL‐1[C458S] (red) and WT (blue) mice aged 0.5, 1.0 and 1.5 years. The number of biological replicates analysed at each age is indicated. The word zero highlighted in blue indicates that no α‐amylase‐resistant, PAS‐positive material could be detected in the WT mice. The error bars show mean ± SEM. Statistical significance between the genotypes was calculated by using two‐way ANOVA and Šidák’s multiple comparison’s test. *Denotes *P* < 0.05, **P < 0.01, ***P < 0.001 and ****P < 0.0001.

Polyglucosan was present in lower amounts in the brain cortex (Fig EV1A and B). It was also present in smaller amounts in the heart, where it reached a maximum level after about a year (Fig 1E and F), and in the lungs and liver (Fig EV1C–F). These results establish that the E3 ligase activity of HOIL‐1 is required to prevent the accumulation of polyglucosan in several tissues.

### HOIL‐1 ubiquitylates glycogen *in vitro*

**Figure 2:**
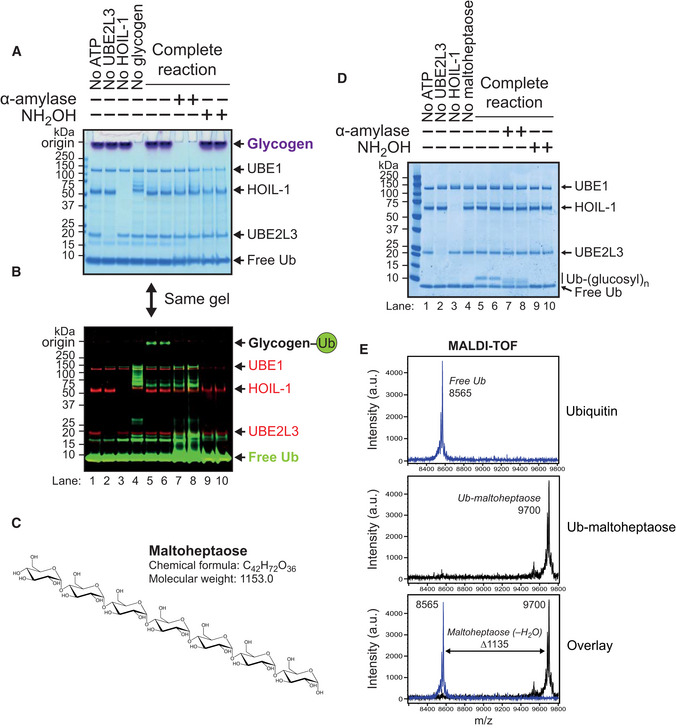
HOIL‐1 ubiquitylates oligo‐ and polysaccharides *in vitro* HOIL‐1 activity towards purified bovine liver glycogen was measured using Cy5‐labelled ubiquitin. Control assays lacking ATP, UBE2L3, HOIL‐1 and glycogen are indicated, as are subsequent treatments with 50 μg/ml human salivary α‐amylase and 1.5 M hydroxylamine (NH_2_OH). The gel has been stained using the periodic acid‐Schiff (PAS) method to visualise glycogen (purple–magenta) and Coomassie Blue stain to visualise protein.Prior to PAS and Coomassie staining, the gel shown in (A) was stained with Flamingo fluorescent protein stain (shown in red) and glycogen ubiquitylation was observed by visualising the fluorescent Cy5‐ubiquitin signal (shown in green).Chemical structure of the α‐1,4‐linked heptasaccharide maltoheptaose.
*In vitro* ubiquitylation of maltoheptaose by HOIL‐1. Assays were treated with α‐amylase or hydroxylamine as indicated. Reaction products were detected by Coomassie staining.Ubiquitylated maltoheptaose was purified from reaction components and analysed by MALDI‐TOF, revealing an additional 1,135 Da attached to the ubiquitin. This is consistent with addition of maltoheptaose to ubiquitin via an oxyester linkage (expected additional mass is 1,153 for maltoheptaose minus 18 to account for the loss of a water molecule during ester bond formation).

We considered how HOIL‐1 might prevent the deposition of polyglucosan and wondered whether HOIL‐1 might not only ubiquitylate the hydroxyl side chains of serine and threonine residues in proteins but also the hydroxyl moieties present in glucose. To investigate whether HOIL‐1 was capable of ubiquitylating glycogen directly, we initially used an *in vitro* fluorescence‐based assay in which commercially available bovine liver glycogen was incubated with HOIL‐1, E1, E2, Mg^2+^‐ATP and fluorescent Cy5‐labelled ubiquitin. The conjugating enzyme UBE2L3 is strictly cysteine reactive and has been reported to be the physiologically relevant E2 conjugating for other RBR ligases, such as HHARI and HOIP (Lewis *et al*, 2015; Dove *et al*, 2017). We therefore included it as the E2 in our assays, as it is also likely to be the cognate E2 that pairs with HOIL‐1. Following SDS‐PAGE, the glycogen (detected by periodic acid‐Schiff [PAS] staining) is too large to enter the gel and remains at the origin (Fig 2A), as does a portion of the fluorescent Cy5‐labelled ubiquitin (Fig 2B, lanes 5 and 6). The comigration of Cy5‐labelled ubiquitin and glycogen required the presence of every reaction component. This high molecular mass Cy5‐ubiquitin signal was lost if the reaction was incubated with α‐amylase to degrade the glycogen (Fig 2B, lanes 7 and 8) or incubated with the oxyester‐specific nucleophile hydroxylamine (Fig 2B, lanes 9 and 10). These experiments indicated that HOIL‐1 had ubiquitylated glycogen and that ubiquitin was attached to it via an oxyester bond.

### Small malto‐oligosaccharides are monoubiquitylated by HOIL‐1 *in vitro*

**Figure EV2:**
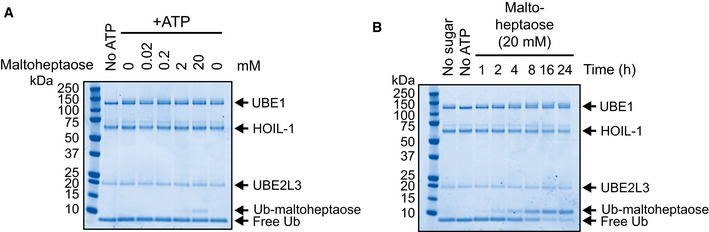
Ubiquitylation of maltoheptaose by HOIL‐1 Increasing concentrations of maltoheptaose were incubated with bacterially expressed HOIL‐1 for 1 h at 37°C and reaction products were resolved by reducing SDS‐PAGE and visualised by Coomassie staining.Maltoheptaose (20 mM) ubiquitylation by HOIL‐1 was assayed for the indicated times and visualised by Coomassie staining.

The majority of the glucose units in glycogen are linked via α‐1,4‐glycosidic bonds. The linear oligosaccharide maltoheptaose contains seven glucose units linked by such bonds (Fig 2C) and was used as a simple model substrate. The replacement of glycogen by maltoheptaose in the *in vitro* ubiquitylation reaction led to the formation of a single more slowly migrating form of ubiquitylated maltoheptaose in a concentration‐ and time‐dependent manner (Fig EV2A and B). As observed for glycogen, this adduct was sensitive to treatment with both hydroxylamine and α‐amylase, although the ubiquitin was still bound to maltose and maltotriose after digestion with α‐amylase because complete hydrolysis to glucose did not take place (Zakowski & Bruns, 1985) (Fig 2D, lanes 7 and 8). Mass spectrometry analysis of ubiquitylated maltoheptaose established that it was a monoubiquitylated species (Fig 2E).

### NMR spectroscopy identifies the hydroxyl group attached to glycosyl carbon C6 as the site of attachment of ubiquitin

**Figure 3:**
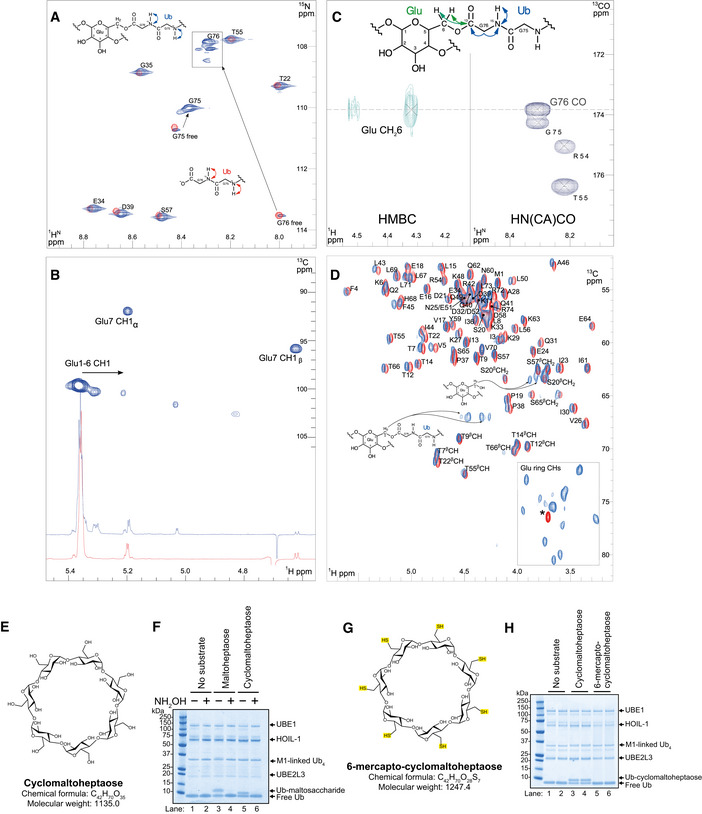
NMR spectroscopy identifies the hydroxyl groups ubiquitylated by HOIL‐1 in maltoheptaose and maltose Glycine region from the ^1^H‐^15^N HSQC spectrum of [^15^N,^13^C] ubiquitin maltoheptaose (blue) overlayed with the same region for [^15^N,^13^C] ubiquitin (red). Visible amide assignments are labelled. Arrows indicate the two major chemical shift changes for Gly75 and Gly76 due to conjugation of maltoheptaose to the C‐terminus.1D ^1^H NMR overlay for the anomeric region of maltoheptaose (red) and [^15^N,^13^C] Ub maltoheptaose (blue) with the ^1^H‐^13^C HSQC of the latter shown above. Key anomeric assignments are labelled, and arrows indicate the chemical shift perturbations upon conjugation. The full aliphatic region of the 1D ^1^H NMR spectra is shown in Fig EV3A.
^1^H‐^13^C HMBC NMR spectrum (left) showing a three‐bond connectivity from the C‐terminal Gly76 carbonyl ^13^C to the 6‐CH_2_ proton chemical shifts. Extracted 2D ^15^N plane (107.9 ppm) from the 3D HN(CA)CO spectrum showing the ^1^HN amide to ^13^C carbonyl correlation for Gly76 of ubiquitin.Overlay of the ^1^H‐^13^C HSQC spectra for free ubiquitin (red) and ubiquitylated maltose (blue). Assignments for ubiquitin CHα proton are labelled together with the assignments for the ligated and free 6‐CH_2_ positions of the disaccharide labelled. Asterisk indicates a buffer component. The full aliphatic region of the 2D ^1^H‐^13^C HSQC spectra is shown in Fig EV3B.Chemical structure of cyclomaltoheptaose (β‐cyclodextrin).HOIL‐1 activity against the indicated maltosaccharides (2 mM) was assayed and visualised by staining with Coomassie protein stain. Where indicated, assays were treated with 1.5 M hydroxylamine. Met1‐linked ubiquitin tetramer was also included in these assays.Chemical structure of 6‐mercapto‐cyclomaltoheptaose (heptakis‐(6‐deoxy‐6‐mercapto)‐β‐cyclodextrin).
*In vitro* ubiquitylation of the indicated cyclodextrins (2 mM) by HOIL‐1, visualised by Coomassie staining. Met1‐linked ubiquitin tetramer was also included in these assays.

To identify the ubiquitin ligation site, a hybrid labelled sample was prepared in which ubiquitin was labelled with NMR isotopes ^15^N and ^13^C and ligated to unlabelled maltoheptaose. The purpose was to reassign ubiquitin and measure NMR connectivity across the protein–carbohydrate interface. As anticipated, the ^1^H‐^15^N HSQC spectrum of [^15^N,^13^C] ubiquitin maltoheptaose shows the characteristic amide peak patterns of ubiquitin and any peak movements could be reassigned readily. Two large amide chemical shift perturbations are observed for the C‐terminal residues G75 and G76, when compared to free ubiquitin (Fig 3A). Strikingly, the amide ^15^N and ^1^HN signals of G76 are shifted upfield by −5.4 ppm and downfield by 0.3 ppm respectively. These values are consistent with those expected for C‐terminal amides when the charged carboxylate is removed by modification (Ulrich *et al*, 2008). No significant chemical shift perturbations are observed elsewhere in ubiquitin. Spin–spin relaxation time (T_2_) of protein ^15^N NMR signals decreases with increasing molecular size. Measured T_2_ values for ubiquitylated maltoheptaose lie in the 130–170 ms range, which agree with those measured for monomeric ubiquitin, compared to T_2_ values of 90–100 ms for diubiquitin. Taken together, these data indicate that a single ubiquitin moiety is conjugated to the oligosaccharide via its C‐terminal glycine residue. Furthermore, the amide of G76 in ubiquitin maltoheptaose exhibits multiple chemical shift environments, with at least four resonances resolved (Fig 3A—boxed), which suggests that different isomers of the conjugate exist with individual maltoheptaose molecules being singly ubiquitylated at various sites along the oligosaccharide.

**Figure EV3:**
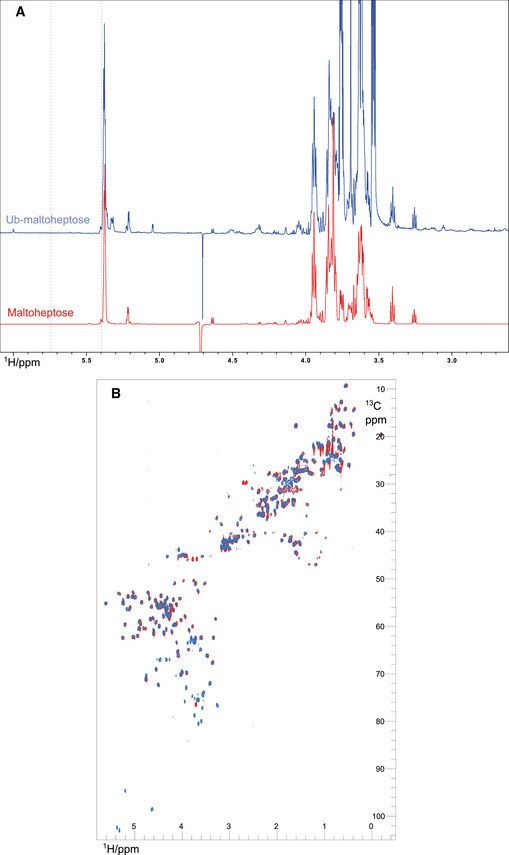
NMR characterisation of ubiquitylated maltoheptaose and maltose Full 1D ^1^H NMR spectra from Fig 3B of maltoheptaose (red) and ubiquitylated maltoheptaose (blue).Complete overlay of 2D ^1^H‐^13^C HSQC NMR spectra from Fig 3D of ubiquitin (red) and ubiquitylated maltose (blue).

Comparison of the anomeric region of the 1D ^1^H NMR spectrum for the ubiquitylated maltoheptaose with free maltoheptaose (Figs 3B and EV3A) shows that while the anomeric protons of the α and β reducing end sugar are largely unaffected, there are several significant chemical shift changes for the anomeric protons of the other glucose residues. As the anomeric position of these sugar residues cannot be targeted by the E3 ligase, the chemical shift changes reflect the effects of ubiquitylation on other positions within these sugars.

To identify unambiguously which position(s) on the glucose units were modified with ubiquitin, we used an ^1^H‐^13^C heteronuclear multiple bond correlation (HMBC) experiment to detect the three‐bond scalar coupling between the sugar protons and the ^13^C carbonyl of G76. These ^3^ *J*_COCH_ couplings are typically 2–3 Hz and correlations in the HMBC would reveal the specific ligated positions. Only one significant correlation could be observed at 4.30 and 4.50 ppm (Fig 3C), corresponding to two protons of a CH_2_ group, and this can be assigned readily using ^1^H correlation NMR spectroscopy to the side‐chain 6‐CH_2_ position of the glucose residues. These chemical shifts match those for 6‐CH_2_ groups typically found in (α1‐6) glucose chains (Dobruchowska *et al*, 2012), thereby confirming the 6‐position modification.

To provide further support for our assignment, we prepared a ubiquitylated maltose sample and compared the natural abundance ^1^H‐^13^C HSQC spectrum with that for free ubiquitin (Figs 3D and EV3B). The 6‐CH_2_ peaks at 4.30 and 4.50 ^1^H ppm (67.0 ^13^C ppm) can be clearly observed in the ubiquitylated maltose sample and not ubiquitin. We also note a minor set of proximal CH_2_ peaks in this region of the spectrum, which could either represent a different conformation of the non‐reducing 6‐CH_2_ group due to restriction in free bond rotation from the attached ubiquitin or some ligation to the reducing end sugar 6‐CH_2_ group, although there are no apparent chemical shift changes for the anomeric proton at the reducing end (Fig 3B). A second 6‐CH_2_ group is also observed (3.85 and 3.90 ^1^H ppm and 63.5 ^13^C ppm in Fig 3D), which corresponds to an unmodified glucose 6‐CH_2_OH group (Bekiroglu *et al*, 2003) and this likely belongs to the reducing end carbohydrate residue of maltose. The ring CH protons are clearly visible between 71 and 81 ppm and these display no significant chemical shift changes when compared to free maltose, suggesting that any ubiquitylation of the ring CHOH groups is absent, or is minimal and beyond detection here.

Consistent with the NMR analysis, we found that cyclomaltoheptaose lacking the reducing C1 hydroxyl and non‐reducing C4 hydroxyl groups could still be ubiquitylated (Fig 3E and F), but the 6‐mercaptan derivative of cyclomaltoheptaose lacking any 6‐CH_2_OH groups could not (Fig 3G and H). It should be noted that the hydroxymethyl (CH_2_‐OH) group of a glycosyl unit is equivalent to the hydroxymethyl side chain of the amino acid serine, a previously identified target for HOIL‐1 ubiquitylation (Kelsall *et al*, 2019b).

Taken together, these experiments show that the single monoubiquitylated species of maltoheptaose observed on SDS gels is a mixture of products, each strictly monoubiquitylated on the C6 hydroxyl moiety but located on distinct single glycosyl units within the oligosaccharide. Since maltoheptaose forms a compact helical arrangement in solution (Goldsmith *et al*, 1982), steric considerations are likely to restrict ubiquitylation to a single site on maltoheptaose. The alternative explanation that ubiquitylation of the first glycosyl residue prevents the ubiquitylation of further glucosyl residues by disrupting the helical structure of maltoheptaose seems less likely, since cyclomaltoheptaose is also singly ubiquitylated, despite being restrained in a pseudohelical conformation by cyclisation that would not be disrupted when the first ubiquitin is added.

### HOIP and Sharpin bind preferentially to polysaccharides with few branch points

**Figure 4:**
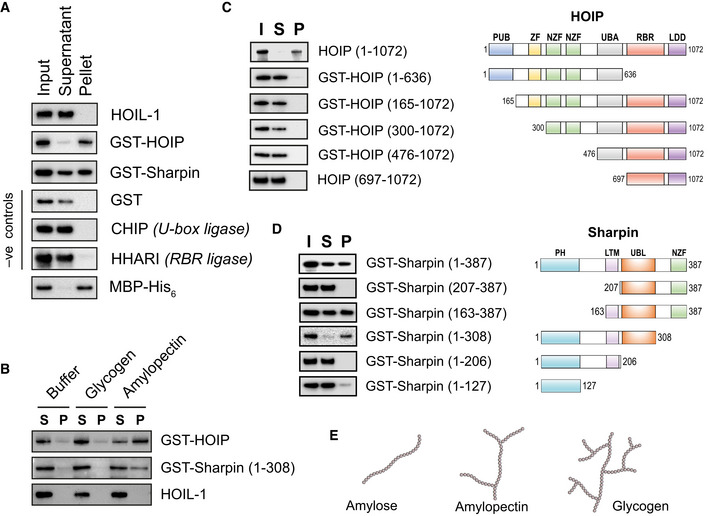
HOIP and Sharpin bind starch‐like glucans *in vitro* One microgram of each of the indicated recombinant proteins was incubated with amylose resin for 60 min, the beads were pelleted by centrifugation and proteins in the pellet (P) and supernatant (S) were compared to the protein input (I) by means of immunoblotting with the appropriate antibodies.Bovine liver glycogen and potato starch‐derived amylopectin were incubated for 60 min with the indicated recombinant proteins, centrifuged for 90 min at 100,000 *g* and protein in the resulting supernatants (S) and pellets (P) analysed by immunoblotting with the appropriate antibodies. Protein without polysaccharide was used as a negative control (Buffer).Amylose‐binding assays of full‐length (1‐1072) and truncated HOIP protein, visualised by immunoblotting. Shown on the right are schematic representations of the truncated HOIP constructs used. Images not drawn to scale. PUB, peptide N‐glycanase and UBA‐ and UBX‐containing protein domain; ZF, zinc finger domain; NZF, Npl4 zinc finger domain; UBA, ubiquitin‐associated domain; RBR, RING‐in‐between‐RING domain; LDD, linear ubiquitin chain determining domain.Amylose‐binding assays of full‐length (1‐387) and truncated Sharpin mutants along with schematic representations of the Sharpin constructs used. Images not shown to scale. PH, Pleckstrin homology domain; LTM, LUBAC‐tethering motif; UBL, ubiquitin‐like domain.Diagrams of amylose, amylopectin and glycogen, indicating differences in polymer chain length and degree of branching.

Since the rate of ubiquitylation of glycogen was slow, taking many hours to convert most of the ubiquitin in the assay to monoubiquitylated maltoheptaose (Fig EV2B), we wondered whether a mechanism(s) might be needed to accelerate the HOIL‐1‐catalysed ubiquitylation of glycogen in cells. We therefore initially investigated the binding of HOIL‐1 and other proteins to the unbranched α1:4‐linked polysaccharide amylose (Fig 4E) to evaluate their glucan‐binding properties (Fig 4A). Unlike maltose‐/maltodextrin‐binding protein (MBP), HOIL‐1 did not bind to amylose–agarose resin, similar to other negative control proteins, such as glutathione S‐transferase (GST). However, to our surprise, the other two components of LUBAC, Sharpin and HOIP did bind to amylose resin, the latter behaving similarly to MBP in the pull‐down assays (Fig 4A). To investigate whether the strength of interaction of HOIP and Sharpin with glucosaccharides depended on the extent of branching, we compared their ability to interact with amylopectin, a polysaccharide component of starch with few branch points, and highly branched glycogen from mammalian cells (Fig 4E). Strikingly, HOIP and Sharpin only bound to amylopectin in these assays (Fig 4B), indicating that these proteins bind preferentially to less branched polyglucosaccharides.

There are no obvious carbohydrate‐binding domains in the primary sequence of either HOIP or Sharpin. We therefore analysed various protein truncations for their ability to bind amylose in our pull‐down assay (Fig 4C and D). None of the truncated HOIP constructs bound to amylose, implying that non‐contiguous regions most likely contribute to glucan binding (Fig 4C). The isolated N‐terminal Pleckstrin homology (PH) domain of Sharpin did not bind to amylose resin, but removal of Sharpin’s Npl4 zinc finger (NZF) domain actually increased binding to amylose (Fig 4D). Constructs containing both the LUBAC‐tethering motif (LTM) and UBL domains of Sharpin interacted with amylose, whereas constructs lacking either of these domains did not (Fig 4D). This suggests that the LTM and UBL domains, known to play important roles in the stabilisation of the trimeric LUBAC (Fujita *et al*, 2018), may participate in binding to unbranched glucosaccharides.

### Allosteric activation of HOIL‐1 by Met1‐linked and Lys63‐linked ubiquitin oligomers

**Figure 5:**
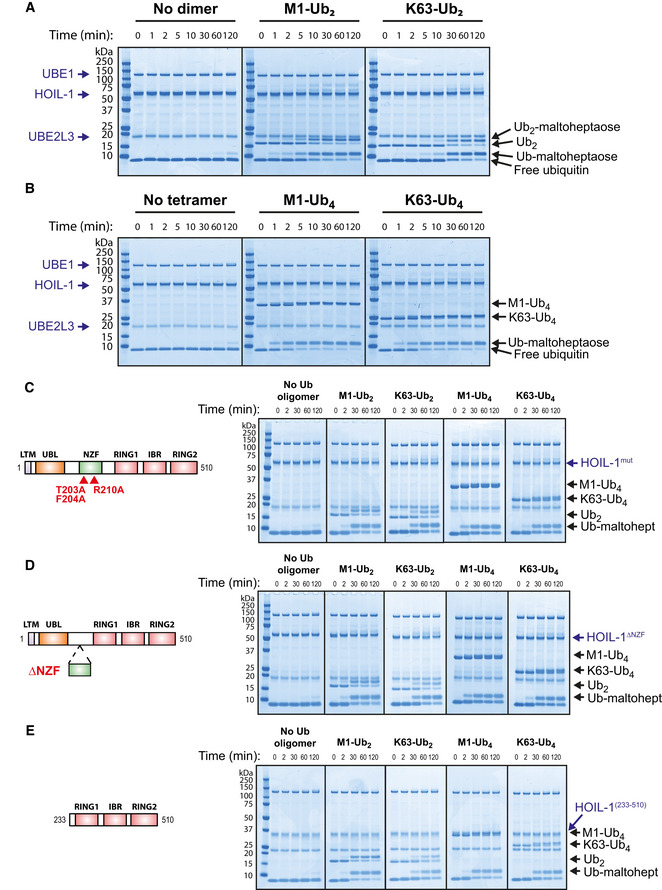
HOIL‐1 is activated allosterically by Met1‐ and Lys63‐linked ubiquitin chains Maltoheptaose ubiquitylation by HOIL‐1 was assayed at 30°C in the presence of Met1‐ or Lys63‐linked ubiquitin dimers for the indicated times and visualised by Coomassie staining.Maltoheptaose ubiquitylation by HOIL‐1 was assayed at 30°C in the presence of Met1‐ or Lys63‐linked ubiquitin tetramers for the indicated times and visualised by Coomassie staining.HOIL‐1[T203A/F204A/R210A] was assayed at 30°C in the absence or presence of the indicated ubiquitin dimers and tetramers. These mutations have been reported to impair the binding of ubiquitin to the NZF domain (Sato *et al*, 2011; Gomez‐Diaz *et al*, 2021) and their approximate location in HOIL‐1 is indicated (schematic not to scale).Assay of HOIL‐1[Δ194‐222] lacking the NZF core domain at 30°C in the absence or presence of the indicated ubiquitin oligomers.Allosteric activation of HOIL‐1[233‐510] (lacking the LTM, UBL and NZF domains at the N‐terminus) by ubiquitin oligomers.

The experiments described in the preceding section suggested a role for HOIP in recruiting HOIL‐1 to glycogen. We therefore wondered whether HOIP might also facilitate the HOIL‐1‐catalysed ubiquitylation of glycogen in other ways. HOIL‐1 contains an Npl4 zinc finger (NZF) domain that is reported to bind M1‐Ub dimers about 10‐fold more strongly than K63‐Ub dimers (Sato *et al*, 2011). We therefore wondered whether M1‐Ub oligomers, formed by the action of HOIP, might bind to the NZF domain of HOIL‐1, inducing a conformational change that accelerated the HOIL‐1‐catalysed ubiquitylation of maltoheptaose. We found that the inclusion of M1‐Ub or K63‐Ub dimers (Fig 5A) or tetramers (Fig 5B) did indeed speed up the rate of conversion of maltoheptaose to ubiquitylated maltoheptaose. Remarkably, after only 1 min, the conversion of ubiquitin to monoubiquitylated maltoheptaose was already greater than after 2 h in the absence of M1‐Ub or K63‐Ub oligomers. After 10–15 min, nearly all the ubiquitin in the assays had been converted to ubiquitylated maltoheptaose (Fig 5A and B). The experiment demonstrated that the rate of ubiquitylation of maltoheptaose was accelerated at least 100‐fold in the presence of small M1‐Ub or K63‐Ub oligomers.

**Figure EV4:**
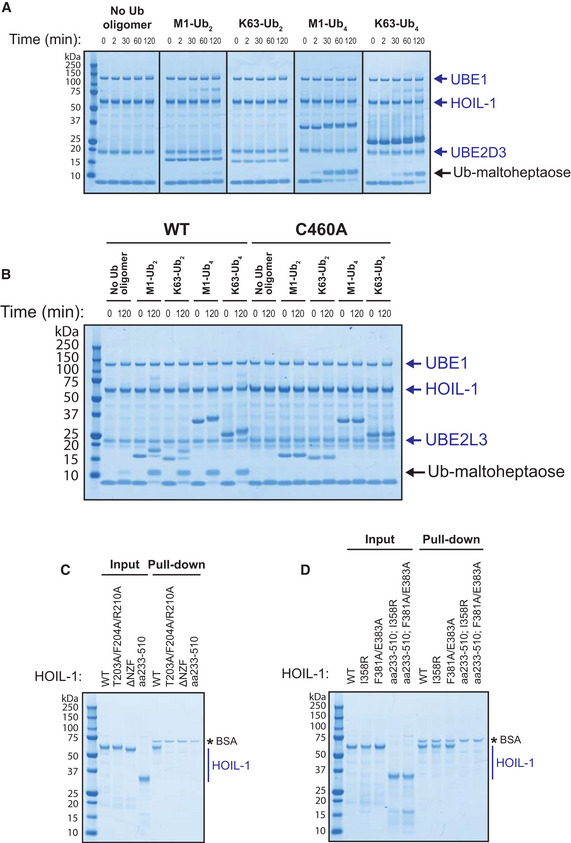
Further characterisation of the allosteric activation of HOIL‐1 by ubiquitin chains Time‐course assays were performed at 30°C with the E2 ubiquitin conjugating enzyme UBE2D3 in the presence of wild‐type HOIL‐1 and the indicated ubiquitin oligomers. Reaction products were detected by Coomassie staining.
*In vitro* ubiquitylation of maltoheptaose by HOIL‐1 or E3 ligase‐inactive HOIL‐1[C460A] was performed for 120 min at 30°C in the presence of the indicated ubiquitin dimers and tetramers. Reaction products were detected by Coomassie staining.Halo‐tagged linear ubiquitin tetramers were covalently coupled to HaloLink resin and pull‐down assays performed with the HOIL‐1 constructs analysed in Fig 5. Bound HOIL‐1 protein was detected by Coomassie staining. * Indicates BSA present in the assay buffer.Halo‐M1‐Ub4 pull‐downs were performed using the HOIL‐1 constructs assayed in Fig 6. * Indicates BSA present in the assay buffer.

The E2‐conjugating enzyme UBE2D3 (UbcH5c) has been reported to permit ester‐linked ubiquitylation when acting in combination with the E3 ligases MYCBP2 and RNF213 (Pao *et al*, 2018; Otten *et al*, 2021). We found that the rate of conversion of ubiquitin to ubiquitylated maltoheptaose was accelerated similarly by M1‐Ub or K63‐Ub oligomers when UBE2L3 was replaced by UBE2D3 (Fig EV4A), although the rate of ubiquitylation was much slower with UBE2D3 (compare Fig EV4A with Fig 5A and B). As expected, no ubiquitylation was observed in the assay with UBE2L3 when HOIL‐1 was replaced by the catalytically inactive HOIL‐1[C460A] mutant (Fig EV4B) (human C460 is equivalent to mouse C458).

To investigate whether the M1‐Ub and K63‐Ub oligomers exerted their effects on HOIL‐1 activity by binding to the NZF domain, we made mutations in this domain that have been reported to impair the binding of M1‐Ub dimers (Sato *et al*, 2011; Gomez‐Diaz *et al*, 2021). Surprisingly, these mutations did not reduce the rate of ubiquitylation of maltoheptaose by either M1‐Ub or K63‐Ub oligomers (Fig 5C). To check this result, we next made a mutant that removed most of the NZF domain, a deletion mutant that is known to abolish the binding of M1‐Ub dimers to the NZF domain (Sato *et al*, 2011). However, the activation of HOIL‐1 by M1‐Ub and K63‐Ub oligomers remained unimpaired (Fig 5D). Moreover, the N‐terminally truncated HOIL‐1[233‐510] mutant, which lacks the LTM, UBL and NZF domains, was also activated by M1‐Ub and K63‐Ub oligomers similarly to wild‐type HOIL‐1 (Fig 5E). Taken together, these experiments revealed that the small Ub oligomers did not activate HOIL‐1 by binding to the NZF domain or any of the other domains located in the N‐terminal region of the protein. They also suggested that HOIL‐1 contained another binding site(s) for M1‐Ub and K63‐Ub oligomers that was most likely located within the RBR domain itself.

**Figure 6:**
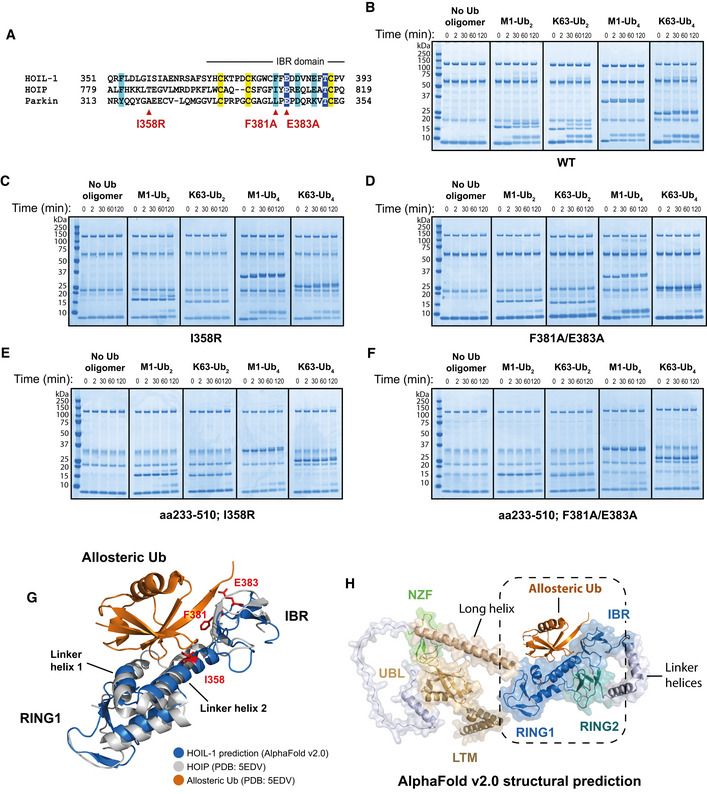
Allosteric ubiquitin binding to the IBR and preceding helical region are important for HOIL‐1 activation Amino acid sequence alignment of HOIL‐1, HOIP and Parkin centred at the start of the IBR domain. Conserved zinc coordinating residues are highlighted in yellow, other highly conserved residues are highlighted with dark blue and weaker conservation of property is indicated by light blue highlight. Red triangles denote the position of the mutations indicated.Time‐course assay performed at 30°C featuring wild‐type HOIL‐1 in the absence or presence of the indicated ubiquitin oligomers, visualised by Coomassie staining.HOIL‐1[I358R], assayed as in (B).HOIL‐1[F381A/E383A], assayed as in (B).The truncated HOIL‐1[233‐510; I358R] mutant was assayed as in (B).The truncated HOIL‐1[233‐510; F381A/E383A] mutant was assayed as in (B).The sequence from RING1‐IBR was aligned by overlaying the HOIL‐1 AlphaFold prediction (residues 282–404, AlphaFold Uniprot ID: Q9BYM8) with residues 699–827 in the crystal structure of HOIP‐RBR (PDB: 5EDV). The location of the allosteric ubiquitin from that HOIP structure is also shown. HOIL‐1 residues I358, F381 and E383 are highlighted in red.Full‐length HOIL‐1 AlphaFold structural prediction (AlphaFold Uniprot ID: Q9BYM8) with likely location of allosteric ubiquitin shown. The region shown in (G) is indicated.

The related RBR E3 ligase family members HOIP and Parkin are activated allosterically by the binding of M1‐Ub dimers (HOIP) and Ser65‐phosphorylated ubiquitin (Parkin) to a ubiquitin‐binding site within the RBR domain (Wauer *et al*, 2015; Lechtenberg *et al*, 2016). We therefore wondered whether the allosteric‐binding site for M1‐Ub dimers and K63‐Ub dimers in HOIL‐1 might be located in the analogous region of the protein and made mutations equivalent to those that impair activation of HOIP and Parkin by M1‐Ub dimers and phospho‐ubiquitin respectively (Fig 6A). Mutation of Ile358 to Arg (equivalent to the Ala320Arg mutation in Parkin that abolishes phospho‐Ub binding) or the double‐mutation Phe381Ala/Glu383Ala (equivalent to combining the Ile807Ala and Glu809Ala mutants that disrupt the allosteric activation of HOIP) resulted in a pronounced reduction or loss of HOIL‐1 activation by Ub dimers, but had less effect on the activation of HOIL‐1 by K63‐Ub tetramers and little effect on ligase activation by M1‐Ub tetramers (Fig 6B–D). We next combined these RBR domain mutations with the HOIL‐1[233‐510] truncation, which ablated the activation of HOIL‐1 by ubiquitin tetramers almost completely (Fig 6E and F). This suggested that, while not essential for the allosteric activation of HOIL‐1, a region(s) N‐terminal to the RBR may contribute to the binding of longer Ub oligomers.

In agreement with the data published using ubiquitin dimers (Sato *et al*, 2011; Gomez‐Diaz *et al*, 2021), we found that the mutation or loss of the NZF domain was sufficient to disrupt the binding of HOIL‐1 to immobilised Halo‐tagged M1‐Ub tetramers (Fig EV4C and D). This indicates that constructs containing the RBR domain, but lacking the NZF domain of HOIL‐1, have a much lower affinity for the M1‐Ub tetramer than constructs containing the NZF domain. This is in keeping with a model in which the binding of M1‐Ub oligomers to the NZF domain of HOIL‐1 anchors LUBAC at its sites of action, whereas the binding of M1‐Ub or K63‐Ub oligomers at the allosteric site on HOIL‐1, which activates this E3 ligase, is weaker and more transient.

Recently, DeepMind and EMBL’s European Bioinformatics Institute created the AlphaFold Protein Structure Database, making freely available structural predictions for all proteins in the human proteome (Jumper *et al*, 2021; Varadi *et al*, 2022). In order to gain more insight into how ubiquitin oligomers influence HOIL‐1 activity, we examined the HOIL‐1 structure predicted in the AlphaFold Protein Structure Database. Alignment of AlphaFold’s HOIL‐1 prediction with the experimentally derived structure of HOIP (Lechtenberg *et al*, 2016) revealed remarkable similarities in the conformations of the RING1 and in‐between‐RING (IBR) domains of these two proteins (Fig 6G), and suggested that the allosteric ubiquitin is accommodated by a common binding site in HOIL‐1 and HOIP (Fig 6G and H). Our mutagenesis data also revealed that hydrophobic interactions with ubiquitin’s Ile44 hydrophobic patch, coupled with the use of a glutamic acid “clamp” to bind the two arginines (Arg72/Arg74) at the C‐terminus of ubiquitin, are employed similarly in both proteins.

### HOIL‐1 can polyubiquitylate maltoheptaose by catalysing the *en bloc* transfer of Ub oligomers directly to the oligosaccharide

**Figure 7:**
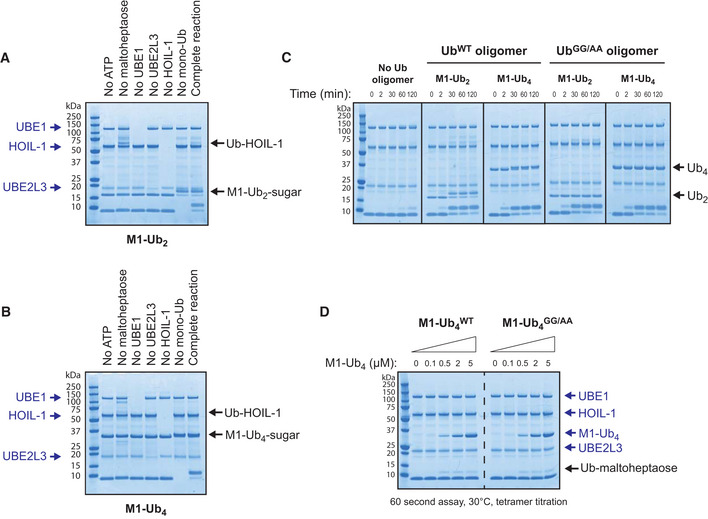
HOIL‐1 catalyses *en bloc* transfer of pre‐formed ubiquitin oligomers directly to maltoheptaose Requirements for *en bloc* transfer of Met1‐linked ubiquitin dimer to maltoheptaose were investigated by leaving out the reaction components indicated. Reaction products were detected by staining with Coomassie.Requirements for *en bloc* transfer of Met1‐linked ubiquitin tetramer to maltoheptaose were investigated by leaving out the reaction components indicated.Time‐course assay performed at 30°C in the presence of Met1‐linked ubiquitin oligomers in which the two most C‐terminal residues were either glycine (normal ubiquitin) or alanine to prevent conjugation to substrates (GG/AA).Maltoheptaose ubiquitylation was assayed for 60 s at 30°C in the presence of the indicated ubiquitin tetramers.

**Figure EV5:**
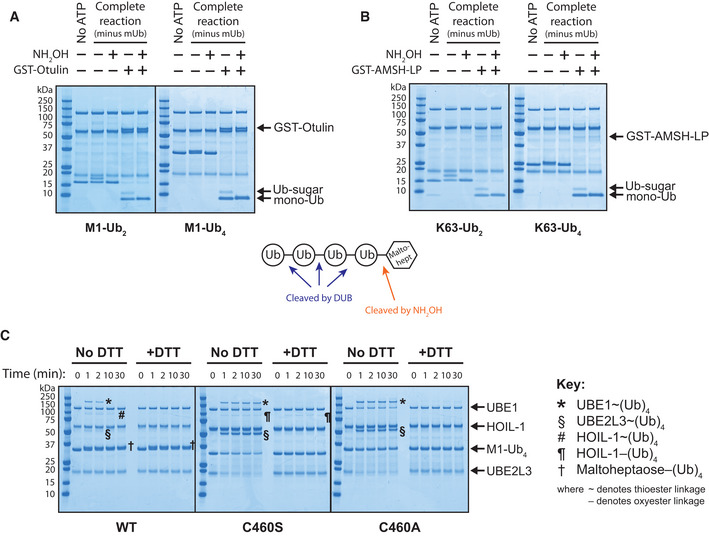
*En bloc* transfer of pre‐formed ubiquitin chains to maltoheptaose proceeds via formation of a thioester intermediate and results in an Otulin and AMSH‐LP‐resistant linkage *En bloc* transfer of Met1‐linked ubiquitin dimers and tetramers was performed for 45 min at 37°C in the absence of monomeric ubiquitin. The reaction was terminated (and further reaction prevented) by addition of the UBE1 inhibitor MLN7243 to a final concentration of 25 μM and incubation for 15 min at 30°C. This was followed by treatment with 1.3 M hydroxylamine and/or 1 μM GST‐Otulin for 60 min at 37°C. Reaction products were separated by SDS‐PAGE and visualised by Coomassie staining.
*En bloc* transfer of K63‐linked ubiquitin dimers and tetramers was performed in the absence of monomeric ubiquitin, followed by inhibition with 25 μM MLN7243 and treatment with 1.3 M hydroxylamine and/or 0.2 μM GST‐AMSH‐LP[264‐436].
*En bloc* transfer of M1‐Ub_4_ to maltoheptaose was performed in the absence of monomeric ubiquitin using wild‐type HOIL‐1 or the catalytically inactive mutants C460S and C460A. Reactions were terminated at the indicated time points by the addition of NuPAGE LDS gel loading buffer without or with 50 mM dithiothreitol (DTT), subjected to SDS‐PAGE and visualised by Coomassie staining. DTT‐sensitive adducts indicate thioesters, whereas those insensitive to reducing agent represent oxyesters, such as that in maltoheptaose−(Ub)_4_ or the engineered oxyester linkage between M1‐Ub_4_ and Ser460 in HOIL‐1[C460S].

Interestingly, during the HOIL‐1‐catalysed reaction, the M1‐Ub or K63‐Ub dimers and tetramers included to accelerate the rate of formation of monoubiquitylated maltoheptaose, themselves became attached covalently to maltoheptaose and at a similar rate to the formation of monoubiquitylated maltoheptaose (Figs 5, 6, and EV4A and B). This *en bloc* transfer of pre‐assembled ubiquitin chains to maltoheptaose was dependent on UBE1 and UBE2L3, and did not require free monoubiquitin (Fig 7A and B). The oxyester bond linking maltoheptaose to the ubiquitin dimer or tetramer was hydroxylamine sensitive and could not be cleaved by the M1‐Ub‐specific deubiquitylase Otulin or the K63‐Ub‐specific deubiquitylase AMSH‐LP (Fig EV5A and B). Performing time‐course assays allowed the visualisation of a dithiothreitol‐sensitive tetraubiquitin adduct on UBE1, UBE2L3 and HOIL‐1 that was prominent at early time points but decreased after 2 min (Fig EV5C left panel). When wild‐type HOIL‐1 was replaced with a catalytically inactive C460S or C460A mutant, the transient adducts on UBE1 and UBE2L3 could not be discharged and persisted, while the use of the C460S mutant similarly stabilised a dithiothreitol‐insensitive oxyester‐linked tetraubiquitin adduct on HOIL‐1 (Fig EV5C, middle and right‐hand panels). These experiments are consistent with the HOIL‐1‐catalysed conjugation of ubiquitin oligomers to maltoheptaose *en bloc*. The HOIL‐1‐initiated polyubiquitylation of glycogen (and proteins) in cells might therefore be catalysed in a single step, and not by HOIL‐1‐catalysed monoubiquitylation followed by the sequential addition of further ubiquitin molecules through the action of other E3 ligases.

The finding that ubiquitin dimers and tetramers were substrates, as well as activators of HOIL‐1, raised the question of whether activation was dependent or independent of their ability to act as substrates. We therefore generated M1‐Ub dimers and tetramers in which the most distal C‐terminal Gly–Gly sequence was mutated to Ala–Ala. These mutants could no longer be conjugated to maltoheptaose, but remained equally potent activators of the HOIL‐1‐catalysed monoubiquitylation of maltoheptaose (Fig 7C). The rate of ubiquitylation of maltoheptaose did not decrease significantly if the M1‐Ub tetramer concentration in the assay was decreased from 5.0 to 0.5 µM at a constant HOIL‐1 concentration of 2.0 µM (Fig 7D), indicating that they were acting catalytically. Taken together, the results support the conclusion that ubiquitin oligomers activate HOIL‐1 allosterically and not by acting as alternative substrates of this E3 ligase.

## Discussion

Ever since the discovery of lysosomal α1:4 glucosidase deficiency (Pompe disease (Hers, 1963)), it has been known that glycogen‐like molecules are continually being transported from the cytosol to the lysosomes, where they are taken up and hydrolysed by lysosomal α1:4 glucosidase to enable the glucose units in glycogen to be recycled. More recently, it has been recognised that this process occurs by a mechanism akin to autophagy, a process in which organelles or pathogenic bacteria are polyubiquitylated prior to their uptake into endolysosomes where they are destroyed (Noad *et al*, 2017; Polajnar *et al*, 2017; van Wijk *et al*, 2017; Tripathi‐Giesgen *et al*, 2021). The uptake of glycogen into lysosomes and its hydrolysis has been termed glycophagy (Jiang *et al*, 2011; Zhao *et al*, 2018), and it has been proposed that starch‐binding domain‐containing protein 1 (Stbd1), a protein localised to perinuclear or lysosomal membranes, may act as a cargo receptor in this process (Jiang *et al*, 2010, 2011).

Glycophagy has generally been considered to be a device for removing normal glycogen from cells to prevent it from accumulating in excess of the cell’s requirements and to enable the excess glucose to be recycled for use by other cells (Zhao *et al*, 2018). However, it is also possible that additional quality control mechanisms exist to remove abnormal forms of glycogen from cells, for example, glycogen molecules with few α1:6 branch points that may be formed adventitiously in trace amounts by errors of metabolism. If not removed rapidly, these starch‐like molecules may precipitate as polyglucosan deposits, which gradually accumulate until they reach levels that cause serious damage to tissue functions as shown, for example, by the fatal diseases that arise in humans with deficiencies in glycogen‐branching enzyme, lysosomal α1:4 glucosidase or HOIL‐1.

Until now, the quality control mechanism by which unbranched glucose polymers are distinguished from normal glycogen has been obscure but, based on the results presented in this study, we suggest that α1:4‐linked oligosaccharides failing to form α1:6 branch points are recognised by the HOIP and Sharpin components of LUBAC enabling the HOIL‐1 component to monoubiquitylate these unbranched glucosaccharides. The monoubiquitylated glucosaccharides may then be polyubiquitylated by other E3 ubiquitin ligases, which might include HOIP, leading to their recognition by the glycophagy machinery and uptake into lysosomes, where they are hydrolysed.

Although we have demonstrated the essential role of HOIL‐1’s E3 ligase activity in preventing polyglucosan deposition *in vivo* and that HOIL‐1 can monoubiquitylate unbranched glucosaccharides at C6 hydroxyl groups *in vitro*, further work is still needed to connect these two findings and provide the additional evidence needed to establish this new aspect of biology. In particular, it will be important in future studies to investigate whether the interaction of native heterotrimeric LUBAC with unbranched glucosaccharides stimulates their ubiquitylation by HOIL‐1 *in vitro*, whether LUBAC is recruited to unbranched glucosaccharides/polyglucosan *in vivo* and, most critically, to investigate whether ubiquitin is attached covalently to unbranched glucosaccharides *in vivo* by the action of HOIL‐1, at least transiently.

In conclusion, our results are the first to describe the ubiquitylation of sugars *in vitro*, and to suggest a potential role for sugar ubiquitylation in cells. Recent work from the Randow laboratory has described the ester‐linked ubiquitylation of bacterial lipopolysaccharide by the E3 ligase RNF213 (Otten *et al*, 2021). However, the authors did not identify the site of ubiquitin attachment, so it is unclear whether it is the lipid or sugar moieties of lipopolysaccharide that undergo ubiquitylation. Nevertheless, their paper and the present study indicate that ester‐linked ubiquitylation of non‐proteinaceous biological molecules may be a tactic employed by more than one family of ubiquitin ligases to enable the ubiquitylation of amine‐free substrates.

## Materials and Methods

### Histological examination of mouse tissues

E3 ligase‐inactive HOIL‐1[C458S] mice (Kelsall *et al*, 2019b) and wild‐type littermates were euthanised by injecting a lethal dose of sodium pentobarbitone. The brain, heart, lungs and liver were removed and fixed for 48–72 h in 10% neutral buffered formalin. Tissues were processed and 3 μm deparaffinised sections were treated for 45 min with 0.5% (w/v) α‐amylase (Sigma), rinsed for 30 min in water and processed for periodic acid‐Schiff (PAS) staining. The tissue sections were assessed by a veterinary pathologist (C.S.) blinded to the genotype of the mice in the different cohorts. A simple semi‐quantitative scoring system was used for the assessment of α‐amylase‐resistant PAS‐positive materials. Scores for amylase‐resistant PAS‐positive deposits were defined as: (i) occasional granular deposits, just detectable by light microscopy; (ii) increased numbers of deposits, readily detectable at ×10 magnification, and (iii) markedly increased numbers of granular deposits easily identifiable by light microscopy. The photomicrographs were captured with Nanozoomer software from whole slide image scans prepared using a Hamamatsu slide scanner.

### Plasmids and proteins

DNA constructs were generated by MRC‐PPU Reagents & Services and sequenced by MRC‐PPU DNA Sequencing & Services (www.dnaseq.co.uk). All plasmids used in this study are available upon request at https://mrcppureagents.dundee.ac.uk. Full‐length untagged human HOIL‐1 (and various mutants thereof) was expressed as His_6_‐SUMO‐HOIL‐1 in BL21 (DE3) *E*. *coli* cells before removal of the tag with the SUMO protease Ulp1 and buffer exchange by size exclusion chromatography or dialysis into PBS, 1 mM TCEP. Human ubiquitin, GST‐Otulin and GST‐AMSH‐LP [246‐436] were expressed and purified as described elsewhere (Ritorto *et al*, 2014). The expression and purification of His_6_‐UBE1 and UBE2L3 (Kelsall *et al*, 2019b), UBE2D3 (Pao *et al*, 2018), GST‐CHIP (Zhang *et al*, 2005) and HHARI (Duda *et al*, 2013) have been described previously. HOIP and Sharpin were expressed in BL21 cells as GST‐tagged fusion proteins, purified on GSH Sepharose and collected by elution with 10 mM glutathione or by removal of the GST tag on the resin using PreScission Protease. MBP‐His_6_ was purified by Ni^2+^ Sepharose chromatography. Untagged Lys63‐linked and linear di‐ and tetraubiquitin chains were produced and purified according to previously reported methods (Komander *et al*, 2008; Dong *et al*, 2011). His‐Halo‐tagged M1‐Ub tetramers were purified by Ni^2+^ Sepharose chromatography and ubiquitin oligomers in which the C‐terminal Gly–Gly sequence of the distal ubiquitin was mutated to Ala–Ala were purified by means of an N‐terminal, TEV protease‐cleavable, DAC tag (Lee *et al*, 2012). All proteins were prepared in PBS, 1 mM TCEP and stored in aliquots at −80°C.

### HOIL‐1‐catalysed ubiquitylation assays using fluorescent Cy5‐labelled ubiquitin

Reactions (20 μl) contained 500 nM His_6_‐UBE1, 2 μM UBE2L3, 2 μM HOIL‐1, 10 μM Cy5‐ubiquitin (South Bay Bio) and 10 mg/ml bovine liver glycogen (Sigma) in phosphate buffered saline pH 7.4 containing 0.5 mM TCEP and 5 mM Mg^2+^‐ATP. Reactions were incubated at 37°C for 1 h with gentle mixing. Where indicated, reactions were then incubated in the presence of 50 μg/ml (~0.1 units) human salivary α‐amylase (Sigma) or 1.5 M hydroxylamine for a further 60 min at 37°C. Reactions were stopped by adding NuPAGE LDS sample loading buffer supplemented with 50 mM DTT, and denatured at room temperature for 10 min. Reaction products were separated by gel electrophoresis on 4–12% Bis–Tris gradient gels using MES SDS running buffer (Invitrogen) and stained with Flamingo fluorescent protein stain (Bio‐Rad). Visualisation of the fluorescent signals was performed using the ChemiDoc MP Imaging System (Bio‐Rad) and ImageJ software (Schneider *et al*, 2012). In the case of assays using glycogen as substrate, these gels were then stained with periodic acid‐Schiff stain using the Pierce Glycoprotein Staining Kit (Thermo Scientific) and Coomassie stained with InstantBlue Protein Stain (Expedeon).

### HOIL‐1‐catalysed ubiquitylation assays (non‐fluorescent)

Reactions (typically 10–20 μl) contained 500 nM His_6_‐UBE1, 2 μM UBE2L3, 2 μM HOIL‐1, 10 μM ubiquitin and 20 mM maltosaccharide substrate (unless otherwise indicated) in phosphate buffered saline pH 7.4, 0.5 mM TCEP and 5 mM Mg^2+^‐ATP. Reactions were incubated at 37°C for 1 h with gentle mixing, unless otherwise stated. Where indicated, reactions were then incubated in the presence of 50 μg/ml (~0.1 units) human salivary α‐amylase or 1.5 M hydroxylamine for a further 60 min at 37°C. Reactions were quenched by addition of NuPAGE LDS sample loading buffer supplemented with 50 mM DTT, and denatured at room temperature for 10 min. Reaction products were separated by gel electrophoresis on 4–12% Bis–Tris gradient gels using MES SDS running buffer and stained with Coomassie InstantBlue Protein Stain before analysis on the ChemiDoc MP Imaging System. Maltoheptaose was purchased from CarboSynth. Maltose was from Sigma. Cyclomaltoheptaose (β‐cyclodextrin) and 6‐mercapto‐cyclomaltoheptaose (heptakis‐(6‐deoxy‐6‐mercapto)‐β‐cyclodextrin) were from AraChem. The UBE1 inhibitor MLN7243 was purchased from Active Biochem.

Where noted, HOIL‐1 activity was stimulated by addition of chain‐type‐specific ubiquitin dimers or tetramers at a concentration of 2 μM. In these experiments, the ubiquitin chains were pre‐incubated for 30 min at 30°C with all other reaction components except ATP. Reactions were initiated by adding Mg^2+^‐ATP to a final concentration of 5 mM. In order to slow the reaction rate and allow better visualisation of the differences between ligase activation in the presence of different chain types, maltoheptaose concentration was dropped to 10 mM and reactions proceeded at the lower temperature of 30°C for the indicated times.

### Preparation of purified Ub maltoheptaose

Ubiquitylation reactions were carried out as described previously and terminated through the addition of 20% (v/v) trifluoroacetic acid (TFA) to a final concentration of 2%. Ubiquitylated maltoheptaose was separated from unconjugated free ubiquitin by reverse‐phase–high‐performance liquid chromatography (RP–HPLC) using a Dionex Ultimate 3000 System. A Thermo BioBasic column (250 × 10 mm) was equilibrated in aqueous buffer containing 20% (v/v) acetonitrile supplemented with 0.1% (v/v) TFA. A flow rate of 2.3 ml/min and an increasing gradient of acetonitrile from 20% to 60% over 60 min were utilised for sufficient separation. Separated fractions were validated using MALDI‐TOF mass spectrometry before being pooled and freeze‐dried. Ubiquitylated maltose was purified similarly.

### MALDI‐TOF mass spectrometry

For MALDI‐TOF sample preparation, a mixture containing a 1:1 ratio of 2% (v/v) TFA and 2,5 dihydroxyacetophenone (DHAP) matrix solution (7.6 mg of 2,5 DHAP in 375 μl 100% (v/v) ethanol and 12 μl of 12 mg/ml diammonium hydrogen citrate) was added to the sample in a 1:1 ratio. One microlitre of the solutions were spotted in duplicate onto an MTP AnchorChip 1,536 Target (Bruker Daltronics). Samples were air dried at room temperature prior to analysis. All spectra were acquired using a Rapiflex MALDI‐TOF mass spectrometer (Bruker Daltronics) equipped with Compass 1.3 control. Recording took place in automatic mode (AutoXecute, Bruker Daltronics), allowing 6–9 s per target spot as described previously (De Cesare *et al*, 2020). Spectra were visualised using FlexControl software and processed by FlexAnalysis software (version 4.0).

### NMR spectroscopy

[*U*‐^15^N,^13^C]‐labelled Ubiquitin was produced by growing transformed *E. coli* BL21 (DE3) cells (New England Biolabs) in M9‐enriched medium supplemented with ^15^NH_4_Cl (1 g/l) and ^13^C‐glucose (2 g/l). All spectra were recorded on a Bruker Avance III HD 800 MHz equipped with triple‐resonance cryoprobe. Purified [*U*‐^15^N,^13^C]‐labelled ubiquitin maltoheptaose conjugate was freeze dried and dissolved in 0.5 ml of 20 mM phosphate buffer pH 6.5, 150 mM NaCl and 10% D_2_O to a final concentration of 0.5 mM. The sequential NMR assignment of ubiquitin was amended using HN(CO)CACB; HNCACB, HN(CA)CO and HNCO triple‐resonance spectra were acquired at 303 K (Cavanagh *et al*, 2007). 2D heteronuclear spectra such as ^1^H‐^13^C heteronuclear multiple bond correlations (HMBC) spectroscopy and heteronuclear single quantum correlation (HSQC) were recorded with standard experiments.

### Glucan‐binding assay

Amylose covalently bound to agarose resin (New England Biolabs) was pre‐incubated with 10 mg/ml bovine serum albumin (BSA) for 30 min with mixing at room temperature to block non‐specific binding. The resin was pelleted by centrifugation (500 *g* for 2 min) and washed three times with 10 mM phosphate buffer, pH 7.4, containing 137 mM NaCl, 1 mM TCEP, 0.01% (v/v) Tween‐20 and 0.01 mg/ml BSA (binding buffer). One microgram of bacterially expressed protein was then incubated with 30 μl of this blocked amylose resin with mixing for 60 min at 4°C in a final volume of 150 μl of binding buffer. Following incubation, the beads were pelleted by centrifugation (500 *g* for 2 min), the supernatant removed and retained, and the resin washed three times with binding buffer before finally resuspending with buffer to the original 150 μl starting volume. Bound proteins were eluted by addition of NuPAGE LDS gel loading buffer and proteins in the supernatant and pellet were analysed by gel electrophoresis followed by immunoblotting using the following antibodies: anti‐HOIP (#SAB2102031) and anti‐HOIL‐1 (#HPA024185) were from Sigma‐Aldrich; anti‐MBP (#E8032S) was from New England Biolabs; HRP‐conjugated anti‐rabbit secondary (#7074) and HRP‐conjugated anti‐mouse secondary (#7076) were from Cell Signaling Technology and HRP‐conjugated anti‐sheep secondary (ab97130) was from Abcam. Polyclonal antibodies against GST (#S902A), CHIP (#S471B) and HHARI (#S622D) were generated by MRC‐PPU Reagents & Services and are available upon request at https://mrcppureagents.dundee.ac.uk.

The binding of bacterially expressed proteins to bovine liver glycogen (Sigma) and potato starch amylopectin (Sigma) was examined as described (Jiang *et al*, 2010). GST‐HOIP, GST‐Sharpin (1‐308) or untagged HOIL‐1 were diluted in 50 mM Tris–HCl, pH 7.5, 150 mM NaCl, 0.1% (v/v) 2‐mercaptoethanol, 0.1% (v/v) Triton X‐100 and subjected to centrifugation at 10,000 *g* for 20 min at 4°C. The supernatant was mixed with the supernatant of glycogen/amylopectin centrifuged under the same conditions. The final concentration of protein was 2.5 μg/ml and glycogen/amylopectin was 0.25 mg/ml in a volume of 0.5 ml. Protein in the presence of buffer alone was used as a negative control. The samples were incubated for 1 h at 4°C with end‐over‐end mixing, and then subjected to ultracentrifugation for 90 min at 100,000 *g* at 4°C. The pellets were resuspended in buffer to the starting volume of 0.5 mL and proteins in the supernatant and pellet were visualised by immunoblotting with the antibodies indicated.

### Pull‐down assays with Halo‐tagged Met1‐linked ubiquitin chains

HOIL‐1 binding to Met1‐linked tetraubiquitin was performed using a modification of previously described methods (Kelsall *et al*, 2019a). 0.1 mg of Halo‐tagged tetraubiquitin was coupled to 0.1 ml of HaloLink resin (Promega) in 1 ml of coupling buffer (phosphate buffered saline pH 7.4, 0.001% NP‐40 and 1 mM TCEP) overnight at 4°C. The beads were washed five times with binding buffer (phosphate buffered saline pH 7.4, 0.001% NP‐40, 1 mM TCEP and 0.01 mg/ml BSA), then 10 μl of coupled beads was incubated with 2 μg of bacterially expressed HOIL‐1 protein in 0.5 ml of binding buffer for 3.5 h at 4°C. The beads were washed five times with phosphate buffered saline pH 7.4, 0.001% NP‐40 and 1 mM TCEP. Bound proteins were separated on 4–12% Bis–Tris gels and Coomassie stained with InstantBlue Protein Stain.

## Author contributions

**Ian R Kelsall:** Conceptualization; Resources; Formal analysis; Supervision; Investigation; Methodology; Writing—original draft; Writing—review & editing. **Elisha H McCrory:** Resources; Formal analysis; Investigation; Methodology; Writing—original draft; Writing—review & editing. **Yingqi Xu:** Formal analysis; Investigation. **Cheryl L Scudamore:** Formal analysis; Investigation; Visualization. **Sambit K Nanda:** Formal analysis; Investigation. **Paula Mancebo‐Gamella:** Formal analysis; Investigation. **Nicola T Wood:** Resources. **Axel Knebel:** Resources. **Stephen J Matthews:** Formal analysis; Supervision; Investigation; Methodology; Writing—original draft; Writing—review & editing. **Philip Cohen:** Conceptualization; Formal analysis; Supervision; Funding acquisition; Writing—original draft; Project administration; Writing—review & editing.

In addition to the CRediT author contributions listed above, the contributions in detail are:

IRK and PC conceived the study. The experiments were performed by CLS and SKN (Figs 1 and EV1), PMG, EHM and IRK (Fig 2), EHM (Fig EV2), YX, SJM and EHM (Fig 3 and EV3) and IRK (Figures 4, 5, 6, 7, EV4 and EV5). AK, IRK and EHM produced bacterially expressed proteins and NTW produced all the DNA constructs. PC, IRK, EHM and SJM wrote the study.

## Disclosure and competing interests statement

The authors declare that they have no conflict of interest. Prof. Philip Cohen is an EMBO Member. This has no bearing on the editorial consideration of this article for publication.

## Supporting information



Expanded View Figures PDF

## Data Availability

This study includes no data deposited in external repositories.
