# Supplementary material for: HOIL‐1 ubiquitin ligase activity targets unbranched glucosaccharides and is required to prevent polyglucosan accumulation
Source: EMBO J. 2022 Mar 11;41(8):e109700. doi: 10.15252/embj.2021109700 (PMC9016349; doi:10.15252/embj.2021109700)
Supplement: Supplementary file 1 — Expanded View Figures PDF [file EMBJ-41-e109700-s002.pdf]

Expanded View Figures

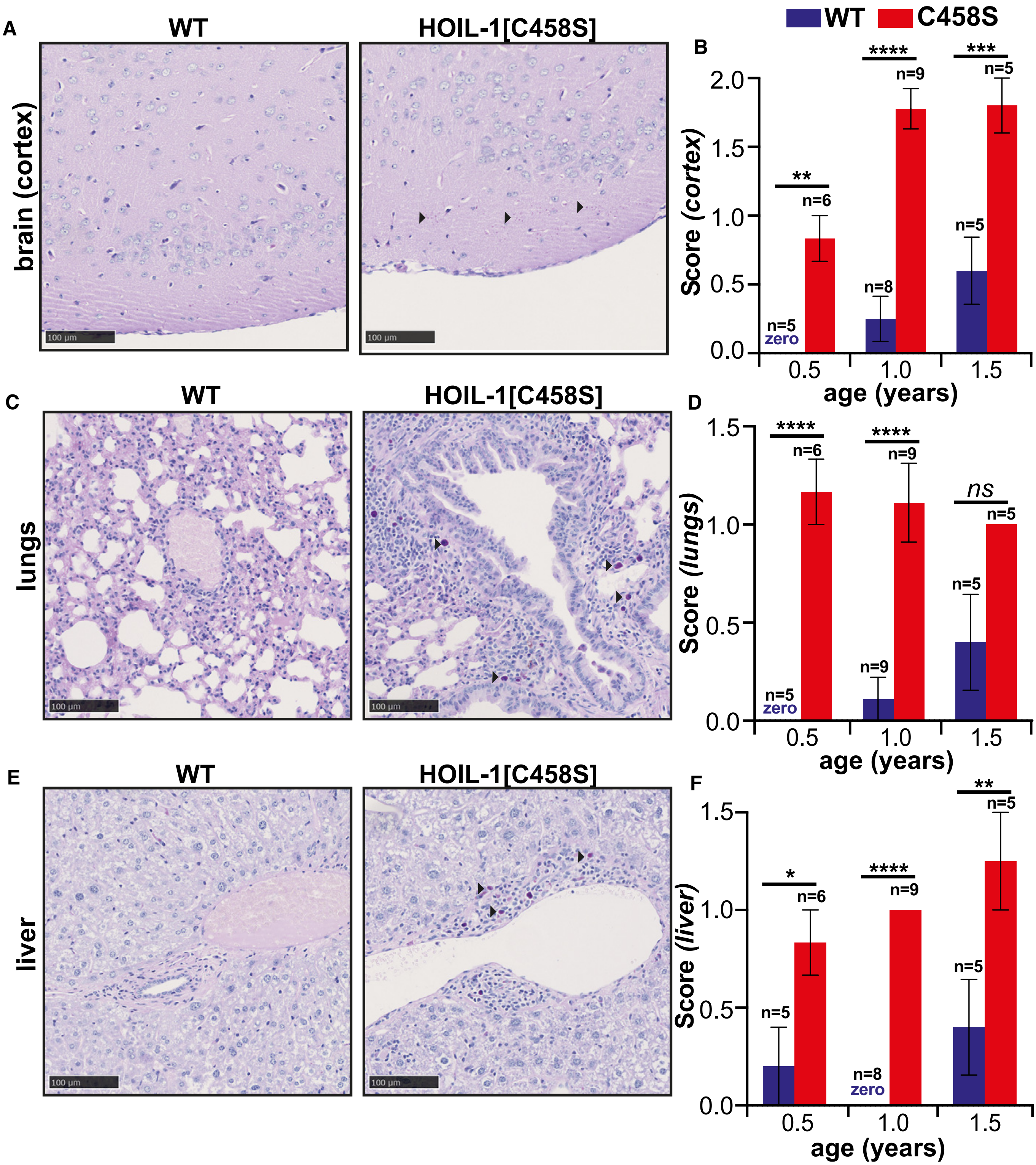

Figure EV1.

©2022 The Authors

Figure EV1. Deposition of α-amylase-resistant polyglucosan deposits in the brain cortex, lungs and liver of HOIL-1[C458S] mice.

A–F Representative PAS-stained sections of the brain cortex (A), lung (C) and liver (E) of 1-year-old HOIL-1[C458S] and WT mice are shown. Scale bar = 100 μm. Arrow heads indicate α-amylase-resistant PAS-positive polyglucosan deposits. Graphs quantitating α-amylase-resistant PAS scores of the brain cortex (B), lung (D) and liver (F) of HOIL-1[C458S] (red) and WT (blue) mice aged 0.5, 1.0 and 1.5 years. The number of biological replicates analysed at each age is indicated. The word zero highlighted in blue indicates that no α-amylase-resistant, PAS-positive material could be detected in the WT mice. The error bars show mean ± SEM. Statistical significance between the genotypes was calculated by using two-way ANOVA and Šidák's multiple comparison's test. *Denotes $P < 0.05$, ** $P < 0.01$, *** $P < 0.001$ and **** $P < 0.0001$.

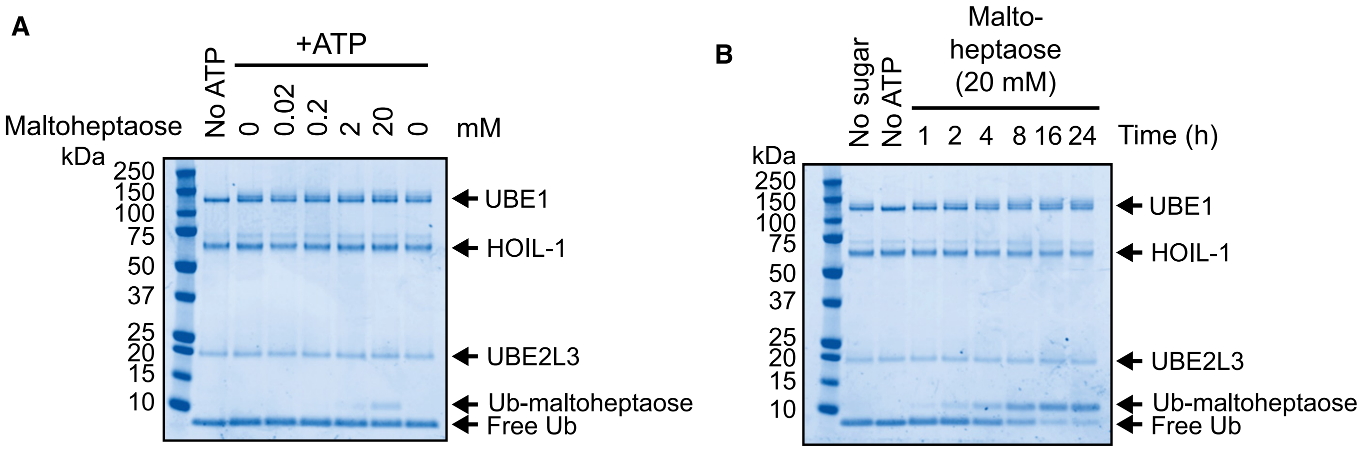

Figure EV2. Ubiquitylation of maltoheptaose by HOIL-1.

A Increasing concentrations of maltoheptaose were incubated with bacterially expressed HOIL-1 for 1 h at 37°C and reaction products were resolved by reducing SDS-PAGE and visualised by Coomassie staining.

B Maltoheptaose (20 mM) ubiquitylation by HOIL-1 was assayed for the indicated times and visualised by Coomassie staining.

Figure EV3. NMR characterisation of ubiquitylated maltoheptaose and maltose.

A Full 1D ^{1}H NMR spectra from Fig 3B of maltoheptaose (red) and ubiquitylated maltoheptaose (blue).

B Complete overlay of 2D ^{1}H-^{13}C HSQC NMR spectra from Fig 3D of ubiquitin (red) and ubiquitylated maltose (blue).

©2022 The Authors

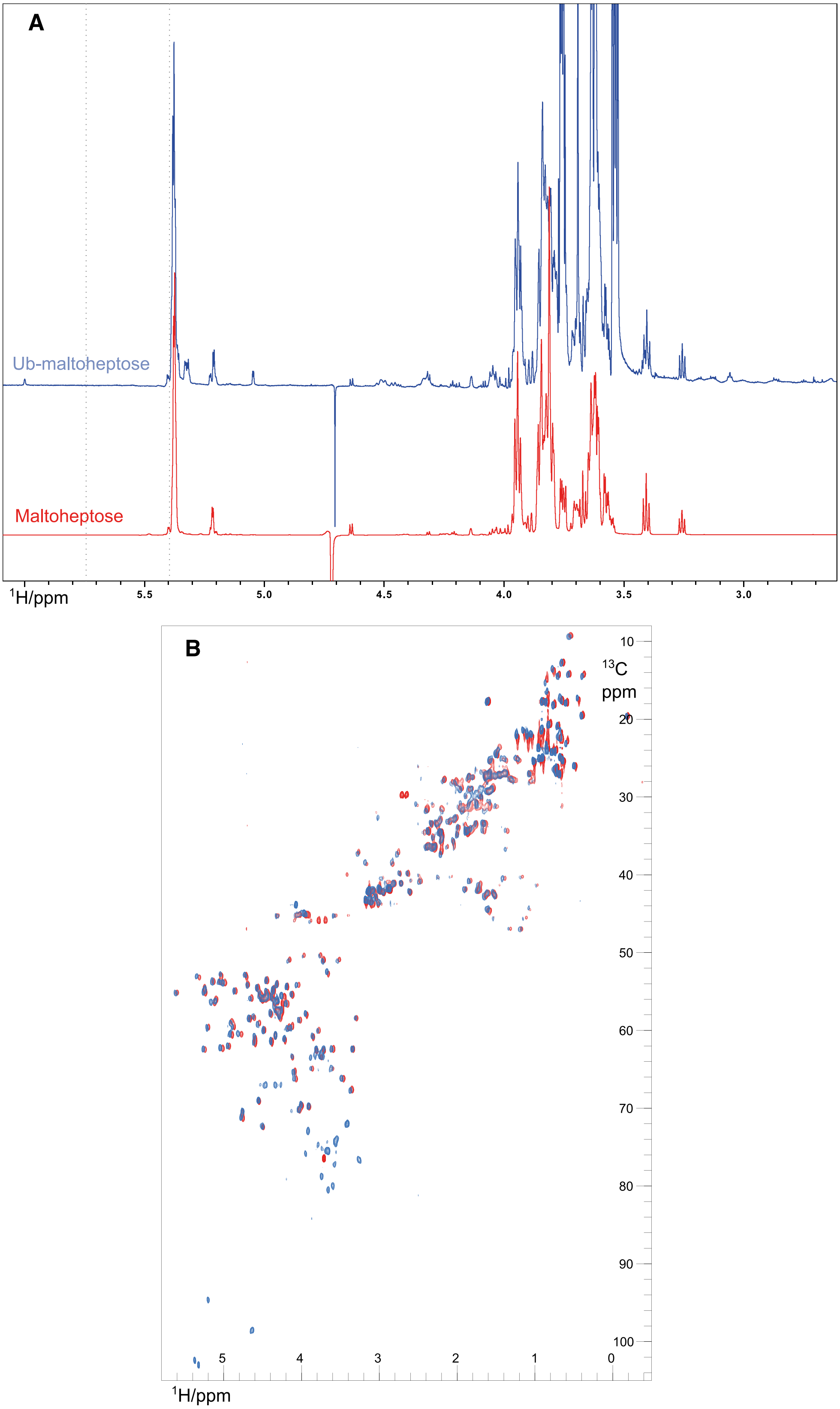

Figure EV3.

 ©2022 The Authors

Figure EV4. Further characterisation of the allosteric activation of HOIL-1 by ubiquitin chains.

A Time-course assays were performed at 30°C with the E2 ubiquitin conjugating enzyme UBE2D3 in the presence of wild-type HOIL-1 and the indicated ubiquitin oligomers. Reaction products were detected by Coomassie staining.

B *In vitro* ubiquitylation of maltoheptaose by HOIL-1 or E3 ligase-inactive HOIL-1[C460A] was performed for 120 min at 30°C in the presence of the indicated ubiquitin dimers and tetramers. Reaction products were detected by Coomassie staining.

C Halo-tagged linear ubiquitin tetramers were covalently coupled to HaloLink resin and pull-down assays performed with the HOIL-1 constructs analysed in Fig 5. Bound HOIL-1 protein was detected by Coomassie staining. * Indicates BSA present in the assay buffer.

D Halo-M1-Ub4 pull-downs were performed using the HOIL-1 constructs assayed in Fig 6. * Indicates BSA present in the assay buffer.

©2022 The Authors

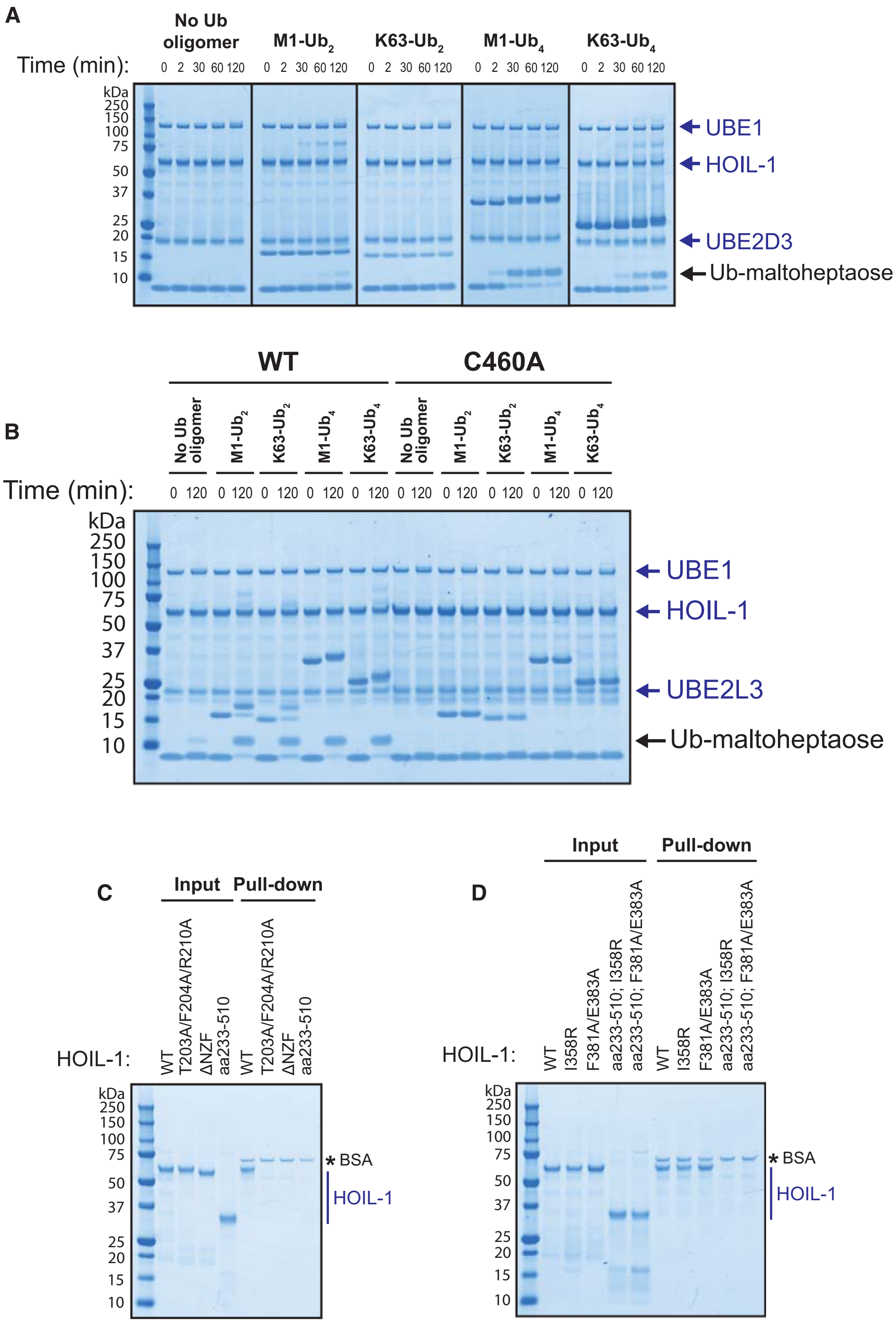
A
No Ub oligomer
M1-Ub2
K63-Ub2
M1-Ub4
K63-Ub4
Time (min): 0 2 30 60 120
kDa
250
150
100
75
50
37
25
20
15
10
UBE1
HOIL-1
UBE2D3
Ub-maltoheptaose
B
WT
C460A
No Ub oligomer
M1-Ub2
K63-Ub2
M1-Ub4
K63-Ub4
Time (min): 0 120
UBE1
HOIL-1
UBE2L3
Ub-maltoheptaose
C
Input
Pull-down
HOIL-1:
WT
T203A/F204A/R210A
ΔNZF
aa233-510
* BSA
HOIL-1
D
I358R
F381A/E383A
aa233-510; I358R
aa233-510; F381A/E383A

Figure EV4.

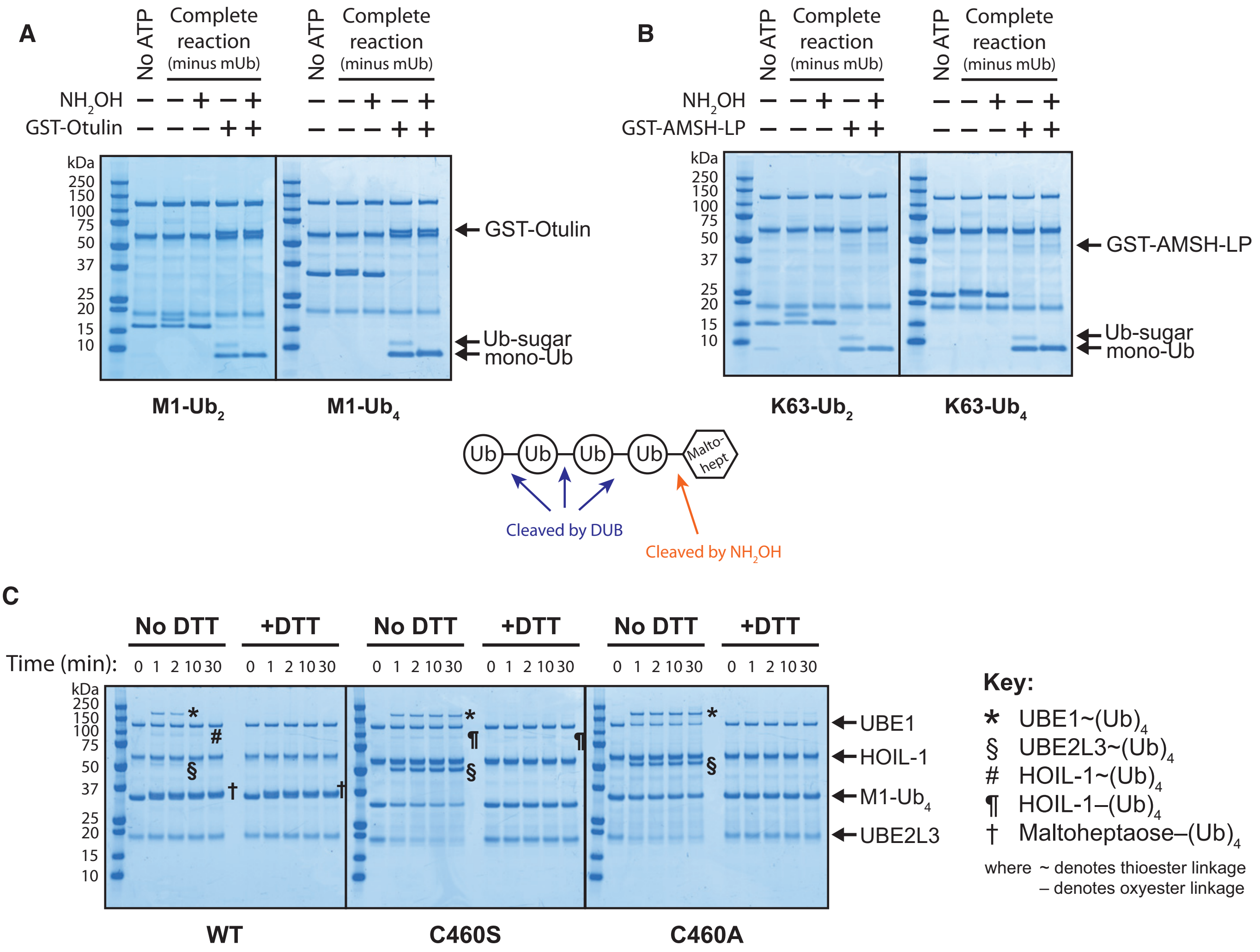

Figure EV5. ***En bloc*** **transfer of pre-formed ubiquitin chains to maltoheptaose proceeds via formation of a thioester intermediate and results in an Otulin and AMSH-LP-resistant linkage.**

A *En bloc* transfer of Met1-linked ubiquitin dimers and tetramers was performed for 45 min at 37°C in the absence of monomeric ubiquitin. The reaction was terminated (and further reaction prevented) by addition of the UBE1 inhibitor MLN7243 to a final concentration of 25 µM and incubation for 15 min at 30°C. This was followed by treatment with 1.3 M hydroxylamine and/or 1 µM GST-Otulin for 60 min at 37°C. Reaction products were separated by SDS-PAGE and visualised by Coomassie staining.

B *En bloc* transfer of K63-linked ubiquitin dimers and tetramers was performed in the absence of monomeric ubiquitin, followed by inhibition with 25 µM MLN7243 and treatment with 1.3 M hydroxylamine and/or 0.2 µM GST-AMSH-LP[264-436].

C *En bloc* transfer of M1-Ub$_4$ to maltoheptaose was performed in the absence of monomeric ubiquitin using wild-type HOIL-1 or the catalytically inactive mutants C460S and C460A. Reactions were terminated at the indicated time points by the addition of NuPAGE LDS gel loading buffer without or with 50 mM dithiothreitol (DTT), subjected to SDS-PAGE and visualised by Coomassie staining. DTT-sensitive adducts indicate thioesters, whereas those insensitive to reducing agent represent oxyesters, such as that in maltoheptaose–(Ub)$_4$ or the engineered oxyester linkage between M1-Ub$_4$ and Ser460 in HOIL-1[C460S].
